# Chromogranin A, Ki-67 index and IGF-related genes in patients with neuroendocrine tumors

**DOI:** 10.1530/EC-13-0052

**Published:** 2013-11-18

**Authors:** R C S van Adrichem, L J Hofland, R A Feelders, M C De Martino, P M van Koetsveld, C H J van Eijck, R R de Krijger, D M Sprij-Mooij, J A M J L Janssen, W W de Herder

**Affiliations:** 1 Department of Internal Medicine, Sector of Endocrinology, Erasmus MC's Gravendijkwal 230, Room D-4303015 CE, RotterdamThe Netherlands; 2 Department of Surgery, Erasmus MCRotterdamThe Netherlands; 3 Department of Pathology, Erasmus MCRotterdam, and Reinier de Graaf HospitalDelftThe Netherlands

**Keywords:** gastroenteropancreatic neuroendocrine tumors (GEP NETs), IGF-related genes, Ki-67 proliferation index, serum chromogranin A (sCgA)

## Abstract

Chromogranin A (CgA) and the Ki-67 proliferation index are considered as important biochemical and pathological markers for clinical behaviour of gastroenteropancreatic neuroendocrine tumors (GEP NETs), respectively. The IGF system has been suggested as an important regulator of GEP NET proliferation and differentiation. A possible relationship between serum CgA (sCgA), Ki-67 proliferation index, and expression of IGF-related genes in patients with GEP NETs has not been demonstrated yet. This study investigates the relationship between sCgA, the Ki-67 proliferation index, and the expression of IGF-related genes in GEP NET tissues and their relation with 5-year survival. Tumor and blood samples from 22 GEP NET patients were studied. Tumoral mRNA expression of IGF-related genes (IGFs: *IGF1*, *IGF2*; IGF receptors: IGF1R, IGF2R; insulin receptors: subtype A (IR-A) and B (IR-B); IGF-binding proteins (IGFBPs): IGFBP1, IGFBP2, IGFBP3, and IGFBP6) was measured using quantitative RT-PCR. Ki-67 proliferation index was determined using immunohistochemistry. sCgA was measured with ELISA. Five-year survival in patients with nonelevated sCgA (*n*=11) was 91 vs 46% in patients with elevated sCgA (*n*=11) (*P*=0.006). IR-A mRNA expression was significantly higher in tumors obtained from patients with elevated sCgA than in those from patients with nonelevated sCgA (6.42±2.08 vs 2.60±0.40; *P*=0.04). This data suggests that sCgA correlates well with 5-year survival of GEP NET patients, and that IR-A mRNA expression correlates well with tumor mass in GEP NET patients.

## Introduction

Gastroenteropancreatic neuroendocrine tumors (GEP NETs) are rare and heterogeneous tumors which may vary according to their biological, functional, and clinical behavior (1). Chromogranin A (CgA) and the Ki-67 proliferation index are considered as important biochemical and pathological markers, respectively, for GEP NET clinical behaviour. The insulin-like growth factor (IGF) system has been suggested as an important regulator of GEP NET proliferation and differentiation (2). Up to present, a possible relationship between serum CgA (sCgA), the cellular expression of the Ki-67 protein, and the IGF-related genes has not been studied in GEP NETs.

Deregulation of the IGF system, a complex network involved in cell growth and metabolic functions in normal tissues and tumors, plays an important role in the pathophysiology of GEP NETs (2). The IGF system consists of different IGF-related genes: two ligands (IGF1 and IGF2), two IGF receptors (IGF1R and IGF2R), two insulin receptors (IR-A and IR-B), and six IGF-binding proteins (IGFBPs). Upon binding to the IGF1R and IR-A, IGFs predominantly generate mitogenic effects. Binding to IR-B predominantly exerts metabolic effects (3, 4). Almost all IGFs are bound to one of the six high-affinity IGFBPs which all differ with regard to their IGF inhibiting and potentiating actions (4, 5, 6, 7). The functions of IGFBP1, IGFBP2, IGFBP3, and IGFBP6 have been well characterized (6).

The Ki-67 proliferation index is generally used for grading of NETs (8, 9, 10). The ENETS/AJCC/WHO 2010 grading system consists of three categories: Grade 1 (G1)=Ki-67 proliferation index ≤2%, G2=Ki-67 proliferation index 3–20%, and G3=Ki-67 proliferation index >20% (8, 9, 11, 12). This grading system has been shown to have relevant prognostic consequences and has been used for decision making with regard to therapeutic options in GEP NET patients (13, 14).

Another important characteristic of GEP NETs is the presence of the CgA protein. CgA is co-secreted by GEP NET cells in the bloodstream with other hormones or peptides (15). CgA is the best available circulating parameter in the follow-up of tumor mass in GEP NET patients (16).

The main aim of our research was to investigate relationships between sCgA levels in GEP NET patients, cellular Ki-67 proliferation index, and the mRNA expression of IGF-related genes in their GEP NET tissues and to correlate this with their 5-year survival.

## Subjects and methods

### Patients with a GEP NET and tissue samples

A total of 22 GEP NET tissue samples from 22 nonconsecutive GEP NET patients were collected before the start of any nonsurgical therapy. The diagnosis of GEP NET was based on clinical, biochemical, radiological, and histopathological characteristics. After tumor excision or biopsy, these tissue samples were immediately frozen in liquid nitrogen and stored at −80 °C. The other tissues were obtained from the Erasmus MC Tissue Bank. These specimens were stored according to a standard procedure (17).

All patients gave written informed consent before inclusion in the studies, which were approved by the Medical Ethics Committee of the Erasmus MC, Rotterdam.

### Biochemical parameters

Blood samples for the determination of sCgA were obtained at the time of diagnosis of the GEP NET (baseline). The sCgA levels were measured using a commercially available ELISA method (CIS Bio International, Gif-sur-Yvette cedex, France; upper limit of normal (ULN) 94 μg/l).

‘Nonelevated’ sCgA was defined as ≤2× the ULN (≤188 μg/l), and ‘elevated’ sCgA was defined as >2×ULN (>188 μg/l). These definitions were based on a previous publication and were selected to maximally exclude other confounding factors which might have caused (slight) elevations of sCgA (18).

### Ki-67 immunohistochemical staining

Immunohistochemical analysis for Ki-67 was performed on 4 μm thick paraffin-embedded tissue sections according to the standardized and optimized benchmark procedure (Benchmark Ultra, Ventana, Tucson, AZ, USA). Pretreatment was performed with CC1 buffer for 64 min at 97 °C. Primary monoclonal mouse antibodies against Ki-67 (clone MIB-1, 1:200 dilution; Dako, Glostrup, Denmark) were incubated for 32 min at 36 °C, and were detected by a high-sensitive detection kit (UltraView Universal DAB Detection kit).

The Ki-67 proliferation index in GEP NET samples was expressed as the percentage of Ki-67 immunopositive NET cells. The counting procedure was performed by three experienced investigators according to the published guidelines (8, 9, 12).

In addition, all GEP NET tissue samples were classified according to the ENETS/AJCC/WHO 2010 grading system: Grade 1 (G1)=Ki-67 proliferation index ≤2%, G2=Ki-67 proliferation index 3–20%, and G3=Ki-67 proliferation index >20% (8, 9).

### Real-time quantitative PCR

For mRNA expression experiments, total RNA of GEP NET tissues was isolated with the ready-to-use High Pure RNA Isolation Kit (Roche Diagnostics). The cDNA synthesis and real-time quantitative PCR (RT qPCR) were performed according to previously published methods (19). Sequences and concentrations of primer-probe sets for all above-mentioned genes are listed in the Supplementary Table 1, see section on supplementary data given at the end of this article. The relative expression of IGF-related genes was calculated using the comparative threshold method, 
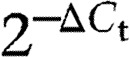
, after efficiency correction of target and reference gene transcripts (*HPRT *) (20, 21).

### Statistical analysis

Data were analyzed using SPSS software (version 17 for Windows; SPSS, Inc.). Comparative statistical evaluations were performed by Mann–Whitney *U* tests. Correlation analysis was accomplished using Spearman's rank correlation tests. Survival rates were calculated using the Kaplan–Meier method, and groups were compared using the log rank test. Kaplan–Meier curves were plotted using overall survival data. The mRNA expression data are reported as mean±s.e.m.

## Results

### Patient characteristics

Patient characteristics are shown in Table 1. Fifty percent of the patients had nonelevated sCgA levels (*n*=11) and the others had elevated sCgA (*n*=11) with median values of 121 and 894 μg/l respectively. As compared to the elevated sCgA group, there were more female patients in the nonelevated sCgA group, these patients were younger, their primary tumor origins were less often in the small intestine and less distant metastases were found.

### Tumor characteristics

In the nonelevated sCgA group, there were four G1 and six G2 patients and, there was one G3 patient. In the elevated sCgA group there were eight G1 and three G2 patients (Table 2).

In the nonelevated sCgA group, four patients were classified as ENETS stage IIIB and the other seven patients were classified as ENETS stage IV. In the elevated sCgA group, two patients were classified as ENETS stage IIIB and the other nine patients were classified as ENETS stage IV.

### Five-year survival of GEP NET patients

**Figure 1:**
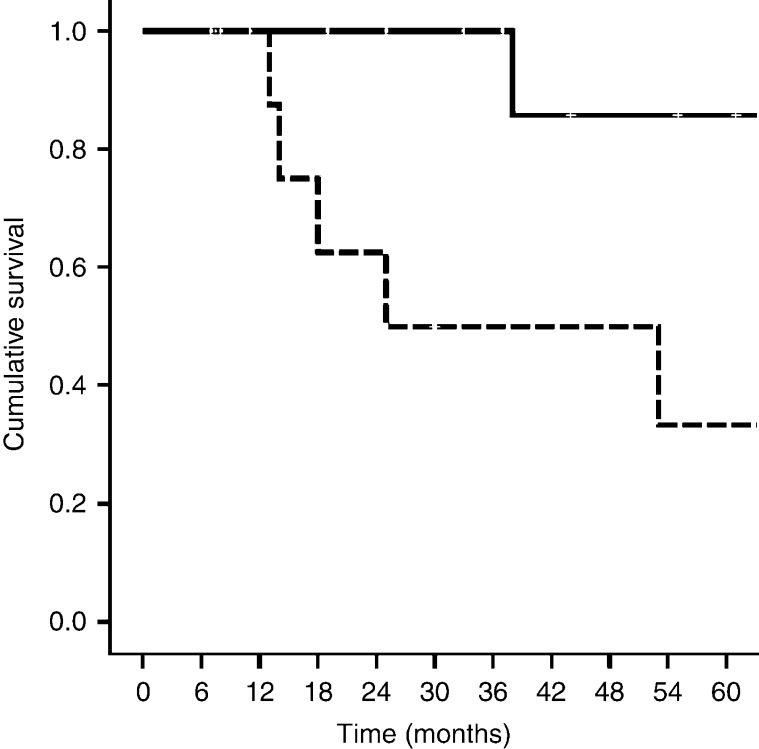
Five-year survival in 22 gastroenteropancreatic neuroendocrine tumor patients (GEP NET) patients divided according to those with nonelevated sCgA (≤2× ULN, *n*=11, solid line) vs elevated (>2× ULN, *n*=11, dashed line; *P*=0.006) sCgA.

In Fig. 1, the 5-year survival of 22 GEP NET patients categorized according to nonelevated and elevated sCgA is shown. There was a significant shorter 5-year survival in the elevated sCgA group as compared with the nonelevated sCgA group (46 vs 91%; *P*=0.006).

In the elevated and nonelevated sCgA groups, no statistical significant correlations could be found between the mRNA expression levels of the different IGF-related genes and 5-year survival. Also, no statistical significant correlation could be demonstrated between the Ki-67 proliferation index and the 5-year survival in these two groups (data not shown).

### Tumoral mRNA expression of IGF-related genes in GEP NET samples

In Table 3, the tumoral mRNA expression levels of IGF-related genes in the nonelevated and elevated sCgA groups are shown. There was a significant higher tumoral mRNA expression for IR-A in the elevated sCgA group compared with the nonelevated sCgA group (2.60±0.40 vs 6.42±2.08, *P*=0.04).

### Correlation between IGF-related genes and Ki-67 proliferation index

No statistical significant relationship between the Ki-67 proliferation index and mRNA expression of IGF-related genes could be demonstrated (data not shown).

## Discussion

To our knowledge, this is the first study in which the relationship between sCgA levels, the tumoral Ki-67 proliferation index, and the tumoral expression of IGF-related genes has been evaluated in GEP NET patients.

Survival analysis showed a significantly shorter 5-year survival in patients with elevated sCgA levels compared with those with nonelevated sCgA levels. sCgA levels generally correlate well with tumor mass. These findings have already been confirmed by other groups (22, 23).

In the elevated and nonelevated sCgA groups, no statistical significant correlations could be found between the mRNA expression levels of the different IGF-related genes and 5-year survival. Also, no statistical significant correlation could be demonstrated between the Ki-67 proliferation index and the 5-year survival in these two groups.

However, other studies have shown a significant shorter survival in Grade 3 GEP NET patients (Ki-67 index >20%) (24, 25). A possible explanation for our discrepant results could be the very small sample size of these heterogeneous tumor entities and the short follow-up.

Our study showed significant higher tumoral mRNA expression of the insulin receptor A (IR-A) in GEP NET patients with elevated sCgA compared with those patients with nonelevated sCgA. Increased expression of the IR-A, a mitogenic variant of the IR, is also found in tumors arising in the colon, breast, thyroid, prostate, and fibrous tissues (26, 27, 28, 29, 30, 31, 32). Until present, these findings have not been reported for GEP NETs.

As sCgA levels correlate well with tumor bulk, our data therefore suggest that tumor mass correlates to tumoral IR-A expression in patients with GEP NETs.

No significant difference in tumoral mRNA expression levels was observed for all other IGF-related genes between patients with nonelevated vs patients with elevated sCgA. Although we have no obvious explanation for these findings, we suggest that IR-A expression has predominantly tumor-stimulating functions in more advanced tumors in contrast to other IGF-related genes, which are involved in the pathophysiology of GEP NETs regardless of the tumor stage.

In conclusion, our study could not demonstrate a relationship between IGF-related genes and the Ki-67 proliferation index in GEP NET tissues. We could confirm previous observations supporting a negative correlation between sCgA levels and 5-year survival. We could not demonstrate a relationship between the tumoral Ki-67 proliferation index and sCgA. However, our study results showed a relation between cellular IR-A mRNA expression and tumor mass.

## Supplementary data

This is linked to the online version of the paper at http://dx.doi.org/10.1530/EC-13-0052.

## Figures and Tables

**Table 1:** Characteristics of 22 GEP NET patients

	**Nonelevated CgA** (≤2× ULN)	**Elevated CgA** (>2× ULN)
Number of patients	11 (50%)	11 (50%)
Sex		
Male	3 (27%)	5 (45%)
Female	8 (73%)	6 (55%)
Age at first diagnosis		
Median (years)	50	59
Range (years)	21–70	47–65
Serum CgA		
Median (μg/l)	121	894
Range (μg/l)	7–176	246–350.800
Primary tumor origin		
Small intestine	7 (64%)	9 (82%)
Pancreas	4 (36%)	2 (18%)

**Table 2:** GEP NET tissue characteristics

	**Nonelevated CgA** (≤2× ULN)	**Elevated CgA** (>2× ULN)
Number of tissues	11 (50%)	11 (50%)
GEP NET tissue		
Primary	9 (82%)	7 (64%)
Small intestine	6	7
Pancreas	3	
Metastasis	2 (18%)	4 (36%)
Lymph node	1	1
Liver	1	3
TNM classification		
T_3_N_1_M_0_	4 (36%)	2 (18%)
T_1_N_0_M_1_		1 (9%)
T_3_N_1_M_1_	7 (64%)	8 (73%)
Grading (Ki-67 index)		
G1 (<3%)	4 (36%)	8 (73%)
G2 (3–20%)	6 (55%)	3 (27%)
G3 (>20%)	1 (9%)	

**Table 3:** Tumoral mRNA expression levels of different IGF-related genes in GEP NET tissue samples of patients with nonelevated and elevated sCgA levels

	**Nonelevated sCgA**	**Elevated CgA**	***P* value**
*IGF1*	0.89±0.32	0.41±0.13	0.30
*IGF1R*	0.26±0.08	0.19±0.04	0.70
*IGF2*	4.10±1.98	2.13±0.90	0.70
*IGF2R*	0.45±0.08	0.67±0.15	0.40
*IR-A*	2.60±0.40	6.42±2.08	0.04
*IR-B*	1.27±0.51	1.26±0.52	1.00
*IGFBP1*	1.50±1.42	1.93±1.17	0.08
*IGFBP2*	7.53±2.22	3.93±1.17	0.22
*IGFBP3*	3.15±1.07	5.40±1.94	0.61
*IGFBP6*	17.50±7.79	21.04±11.25	0.52
